# C-Reactive Protein Levels in relation to Incidence of Hypertension in Chinese Adults: Longitudinal Analyses from the China Health and Nutrition Survey

**DOI:** 10.1155/2021/3326349

**Published:** 2021-12-10

**Authors:** Bo Chen, Yuze Cui, Mengyun Lei, Wenlei Xu, Qiongjie Yan, Xiaotong Zhang, Minghui Qin, Shaoyong Xu

**Affiliations:** ^1^Evidence-Based Medicine Centre, Office of Academic Research, Xiangyang Central Hospital, Affiliated Hospital of Hubei University of Arts and Science, Xiangyang, Hubei, China; ^2^Department of Endocrinology, Xiangyang Central Hospital, Affiliated Hospital of Hubei University of Arts and Science, Xiangyang, Hubei, China; ^3^Department of Nephrology, Xiangyang Central Hospital, Affiliated Hospital of Hubei University of Arts and Science, Xiangyang, Hubei, China; ^4^Department of Traditional Chinese Medicine, Xiangyang Central Hospital, Affiliated Hospital of Hubei University of Art and Science, Xiangyang, Hubei, China

## Abstract

**Objective:**

To explore the association between high sensitivity C-reactive protein (hs-CRP) levels and incident hypertension, as well as the association between hs-CRP levels and related covariates, in a Chinese adult population.

**Methods:**

This study was based on the China Health and Nutrition Survey, a continuing open, large-scale prospective cohort study. Adult participants who were free of hypertension were included at baseline survey in 2009 and were followed up in 2015 (follow-up rate: 77.45%). The hs-CRP was measured using the immunoturbidimetric method and divided into three groups: low-risk group (0 ≤ hs-CRP <1 mg/L), average-risk group (1 ≤ hs-CRP <3 mg/L), and high-risk group (3 ≤ hs-CRP ≤10 mg/L). Definite diagnosis of hypertension in the follow-up survey in 2015 was the endpoint event of this study. The areas under the curve (AUC) of the receiver operating characteristic (ROC) curve analyses were used to evaluate the predictive value of the hs-CRP.

**Results:**

3794 participants were finally included as study sample, of whom 912 developed hypertension during a 6-year follow-up period (incidence: 24.1%). The incidences of hypertension in hs-CRP low-risk, average-risk, and high-risk groups were 17.6% (200/1135), 25.9% (521/2015), and 29.7% (191/644), respectively. Spearman's correlation analyses showed that there was significant positive correlation between hs-CRP levels and waist circumference, total triglycerides, total cholesterol, age, body mass index, and homeostasis model assessment of insulin resistance index. Stepwise regression analyses showed that participants in the hs-CRP high-risk group had a 46.2% higher risk of developing hypertension compared with those in the hs-CRP low-risk group (odds ratio: 1.462, 95% confidence interval: 1.018–2.101). Baseline systolic and diastolic blood pressure levels and waist circumference contributed the most to the development of hypertension with *R*^2^ of 0.076, 0.052, and 0.039, respectively, while hs-CRP had lower area under the curve (AUC) for hypertension, adding baseline BP and WC to the prediction model increased the AUC to 0.708 (95% CI: 0.681–0.735).

**Conclusion:**

This study revealed a weak positive association between CRP levels and future incidence of hypertension in the Chinese population. The combination of hs-CRP with baseline BP and waist circumference (WC) had a higher predictive value for hypertension (AUC: 0.708), but the predictive value was still limited.

## 1. Introduction

Hypertension is one of the three major risk factors, along with smoking and overweightness shared by all chronic noncommunicable diseases (NCDs), and is a condition that increases the burden of disease death [1, 2]. According to studies, in 2010, 31.1% of the global adult population (1.39 billion) suffered from hypertension and its prevalence continues to increase worldwide, especially in low- and middle-income countries [3, 4]. As a common chronic disease and a modifiable risk factor for cardiovascular disease [5], the prevention and control of hypertension should be a major public health priority worldwide.

The role of inflammation in the progression of hypertensive diseases has gained increasing attention, and hypertension has been considered a type of inflammatory disease [6]. An important clinical inflammatory marker is C-reactive protein (CRP). The association between CRP and risk of hypertension has been reported in several cross-sectional and cohort studies [6–16]. Most of these studies support the association between CRP and incident hypertension; for example, Sung et al. confirmed CRP as an independent predictor of hypertension risk based on a retrospective cohort study [8]. However, other studies did not observe an association between baseline CRP and the occurrence of hypertension, suggesting there may not be a significant association [17, 18]. Therefore, it is necessary to continue to study the relationship between CRP and hypertension.

Previous studies have shown that CRP levels can vary across ethnic groups; for instance, African populations have overall higher CRP levels than white populations, and Asian populations seem to have lower CRP [19]. Existing association studies between CRP and the risk of developing hypertension have mainly focused on White populations, a few non-Chinese Asian populations [8, 17], and Chinese populations that focused on the study for individual regions and race [12, 20–22]; however, there have been no large nationwide cohort studies in China. In addition, the available studies largely failed to comprehensively explore the contribution of other confounding factors, such as obesity, blood pressure (BP), and insulin resistance. The complex relationship between CRP and hypertension may be better understood by investigating the contribution of these confounding factors to the pathogenesis of hypertension.

Therefore, the aim of our study was to explore the association between high sensitivity CRP (hs-CRP) levels and incident hypertension, as well as the association between CRP levels and related covariates, in a Chinese adult population.

## 2. Methods

### 2.1. Study Design

The study population for this research was derived from the China Health and Nutrition Survey (CHNS), a continuing open, large-scale prospective cohort study [23], which aims to obtain data from provinces with large variations in geography, economic development, and health status, to explore the impact of China's socioeconomic transition on nutrition and health status of China's population. Currently, there are ten sets of CHNS data (1989, 1991, 1993, 1997, 2000, 2004, 2006, 2009, 2011, and 2015), each of which uses a multistage random sampling method to collect data in each province. The prefectures and municipalities of the nine provinces are stratified by income level (low, middle, and high); a weighted sampling scheme was used to randomly sample four counties in each province. CHNS scientific principles and design are described in detail elsewhere [24, 25]. CHNS was supported by grants from the University of North Carolina Chapel Hill and the China National Institute of Nutrition and Food Safety. CHNS was also approved by the Institutional Review Boards of the Chinese Center for Disease Control and Prevention (CDC) and the China-Japan Friendship Hospital. Participants provided written informed consent prior to the study.

### 2.2. Study Population

**Figure 1:**
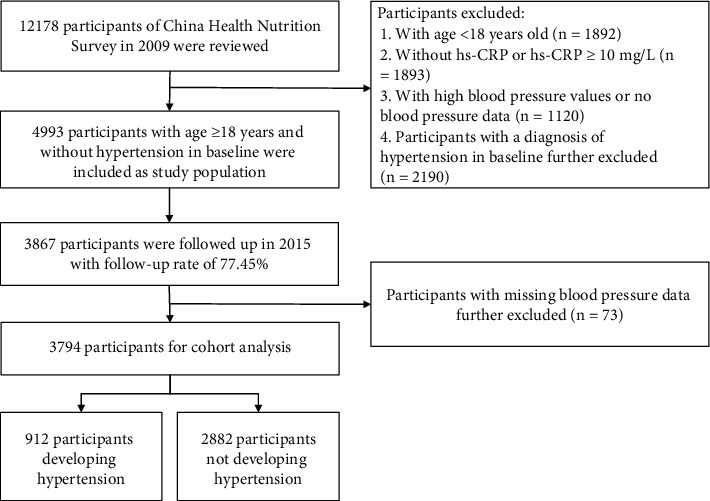
Participant flowchart.

Since the CHNS examined blood samples from participants only in 2009, our study used that dataset as baseline information. 12,178 people participated in the baseline survey in 2009; we excluded 1,892 participants with age <18 years, 1,983 participants without hs-CRP data or hs-CRP ≥ 10 mg/L (indicating acute inflammation) [26], and 1,120 participants with high blood pressure values (systolic blood pressure (SBP) ≥ 140 mmHg or diastolic blood pressure (DBP) ≥ 90 mm Hg) or no blood pressure data. We further excluded 2,190 participants with a diagnosis of hypertension (BP ≥ 140/90 mm Hg or a history of hypertension) at baseline. Of the remaining 4993 participants, 3867 participants attended the follow-up survey in 2015 (follow-up rate: 77.45%). Ultimately, after excluding 73 subjects with missing blood pressure data at follow-up survey, 3794 subjects were included as study samples for analysis (Figure 1).

### 2.3. Data Collection

In 2009, a structured questionnaire was used to collect participant information, including demographic data, socioeconomic characteristics, lifestyle, and general health status, and medical history information of hypertension. Physical examination and blood samples were obtained using standard protocols.

#### 2.3.1. Physical Examination and Biochemical Parameters

Participants were physically examined according to standard procedures. Height was measured to the nearest 0.1 cm using a SECA measuring tape. Weight was measured using a calibrated beam balance and to the nearest 0.1 kg. Body mass index (BMI) (kg/m^2^) = square of weight (kg)/height (m) was determined. Waist circumference (WC) was measured using a SECA measuring tape to the nearest 0.1 cm. After 5 min of sitting, BP was measured using a mercury sphygmomanometer for three consecutive times separated by 3–5 min; the average of three BP measurements was used.

Blood samples were collected via venipuncture after fasting overnight (for at least 8 h) and transferred to a local hospital for processing within 2 h of collection, based on strict quality control standard specifications. All blood samples were analyzed at the National Central Laboratory in Beijing (medical laboratory accreditation certificate: ISO 15189: 2007) [27]. Methods for the determination of total cholesterol (TC), total triglycerides (TG), high-density lipoprotein cholesterol (HDL-C), low-density lipoprotein cholesterol (LDL-C), CRP, and blood glucose are described elsewhere [27]. Briefly, the hs-CRP was measured using the immunoturbidimetric method (Hitachi 7600 automated analyzer, Hitachi, Tokyo, Japan) with Denka Seike (Tokyo, Japan) reagents.

#### 2.3.2. Main Study Variables and Endpoint Events

The graph showing the distribution of hs-CRP is presented by box plots (see Supplementary Figure 1). hs-CRP levels were divided into three groups: low-risk group (0 ≤ hs-CRP < 1 mg/L), average-risk group (1 ≤ hs-CRP < 3 mg/L), and high-risk group (3 ≤ hs-CRP < 10 mg/L) [26, 28]. Definite diagnosis of hypertension in the follow-up survey in 2015 was the endpoint event of this study. Hypertension was defined as meeting at least one of the following criteria: (1) SBP ≥140 mmHg, (2) DBP ≥90 mm Hg, or (3) self-reporting having been diagnosed with hypertension or currently on oral antihypertensive medication during follow-up [29].

#### 2.3.3. Covariates

Subjects were classified as lean (BMI <18.5 kg/m^2^), normal (18.5 kg/m^2^ ≤ BMI <24.0 kg/m^2^), or overweight/obese (BMI ≥24.0 kg/m^2^) according to the criteria recommended by the Chinese Working Group on obesity [30]. Level of physical activity was estimated using metabolic equivalent (MET), obtained by multiplying the time spent per activity with the specific MET value [31]. Homeostasis model assessment of insulin resistance (HOMA-IR) was calculated as follows: HOMA-IR = fasting insulin (mIU/L) × fasting plasma glucose (mmol/L)/22.5. Other covariates, such as urban *vs.* rural area, age, sex, ethnicity (Han Chinese or other), education level, alcohol consumption (yes or not), and smoking (yes or not) were recorded using a structured questionnaire.

### 2.4. Statistical Analysis

Descriptive analyses of continuous variables were reported as mean ± standard deviation (SD) or medians (interquartile range), categorical variables were described by frequency (percentage), and statistical differences of variables in hs-CRP among groups were tested using analysis of variance or Kruskal–Wallis test or chi-square test (Cochran–Armitage or Mantel–Haenszel test), respectively.

We explored the association between baseline hs-CRP levels and other covariates using Spearman's correlation analysis. To explore the association of hs-CRP and risk of hypertension, we first examined the association of the relevant variables with hypertension using a univariate logistic regression model. After obtaining the contribution of each variable to hypertension, we used stepwise logistic regression models to estimate adjusted odds ratios (OR) and 95% confidence intervals (CI). Model 1 did not use any adjustment. Model 2 was adjusted for socioeconomic factors (urban *vs.* rural, age, sex, nationality, and education). Model 3 was further adjusted for lifestyle factors (length of sleep, smoking status, alcohol consumption, physical activity, and total energy intake). After analyzing the collinearity between these blood parameter variables (see Supplementary Table 1), model 4 further adjusted the blood parameters (baseline systolic and diastolic BP, TC, TG, and HDL-C); model 5 was further adjusted for BMI and WC. The areas under the curve (AUC) of the receiver operating characteristic curve (ROC) analyses were used to evaluate the predictive value of the hs-CRP. Stepwise adjusted Cox proportional hazards regression was used to assess robustness as a sensitivity analysis. All statistical tests were 2-sided and performed using SAS 9.4 (SAS Institute, Cary, NC). *p* < 0.05 was considered statistically significant.

## 3. Results

### 3.1. Basic Characteristics

**Figure 2:**
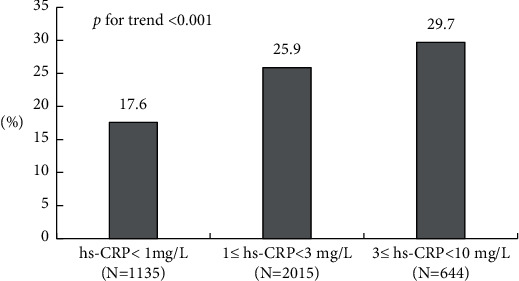
When subjects were divided into three categories based on the baseline hs-CRP levels, a trend relationship between hs-CRP levels and incident hypertension was observed (*p* < 0.001).

3794 participants were finally included as the study sample, of whom 912 developed hypertension during a 6-year follow-up period. The incidences of hypertension in hs-CRP, low-risk, average-risk, and high-risk groups were 17.6% (200/1135), 25.9% (521/2015), and 29.7% (191/644), respectively. Basic characteristics of the study population are shown in Table 1. There were no significant differences between hs-CRP groups for ethnic origin, urban *vs.* rural area, sex, sleep duration, smoking or drinking habits, and physical activity (*p* > 0.05). In addition, those in hs-CRP high-risk group were more often elderly, less educated, had lower HDL-C levels, and had higher levels of BMI, WC, systolic and diastolic BP, as well as higher concentrations of TG, TC, glucose, and LDL-C (*p* < 0.05). A trend relationship between the serum levels of hs-CRP and incident hypertension was observed (*p*< 0.001; Figure 2).

### 3.2. Correlation of hs-CRP with Relevant Covariates

The baseline data were used to analyze the correlation between hs-CRP and related covariates (Table 2). There was a significant positive correlation between hs-CRP (ordinal variable) and WC, *r* = 0.243 (95% CI: 0.212–0.273); hs-CRP was significantly correlated positively with TG, TC, age, BMI, SBP, DBP, and HOMA-IR index (*p* < 0.001) with a correlation coefficient of 0.211 (95% CI: 0.180–0.241), 0.184 (95% CI: 0.153–0.215), 0.189 (95% CI: 0.158–0.219), 0.186 (95% CI: 0.155–0.216), 0.133 (95% CI: 0.099–0.167), 0.104 (95% CI: 0.071–0.389), and 0.152 (95% CI: 0.121–0.183); hs-CRP *vs.* HDL-C showed a significant negative correlation, *r* = −0.154 (95% CI: −0.184∼−0.122) (Table 2). hs-CRP was not significantly associated with urban *vs.* rural area, sex, ethnicity, physical activity, alcohol consumption, or sleep duration (*p* > 0.05).

### 3.3. Associations between Variables and Hypertension

The results of the univariate analysis are shown in Table 3. Compared to the hs-CRP low-risk group, ORs for the development of hypertension in the hs-CRP average-risk group and hs-CRP high-risk group were 1.630 (95% CI: 1.359–1.956) and 1.971 (95% CI: 1.570–2.476), respectively. ORs for the association of baseline systolic and diastolic BP levels with the development of hypertension were 1.052 (95% CI: 1.044–1.061) and 1.039 (95% CI: 1.032–1.045), respectively, with *R*^2^ = 0.076 and 0.052, respectively. Among socioeconomic factors, age contributed the most to the incidence of hypertension (*R*^2^ = 0.063). OR for the association between age at baseline and incident hypertension was 1.039 (95% CI: 1.032–1.045, *R*^2^ = 0.063). OR for baseline WC and incident hypertension was 1.040 (95%CI: 1.032–1.048, *R*^2^ = 0.039). Compared with those in the lowest group of BMI (BMI <18.5 kg/m^2^), the ORs for the development of hypertension in the 18.5 kg/m^2^ ≤ BMI <24 kg/m^2^ group and ≥24 group were 1.183 (95% CI: 0.880–1.591) and 2.127 (95% CI: 1.575–2.874), respectively, and *R*^*2*^ = 0.024 (Table 3).

Logistic regression was used to analyze the risk of incident hypertension with hs-CRP. hs-CRP grouping levels were positively associated with incident hypertension (*p*<0.05). Participants in the hs-CRP high-risk group had a 46.2% higher risk of developing hypertension compared with those in the hs-CRP low-risk group in Model 5 after adjustment for all covariates (OR: 1.462, 95% CI: 1.018–2.101; *p* = 0.03) (Table 4). Logistic regression analysis revealed that the associated factors in Model 2 contributed the most to the risk of developing hypertension (*R*^2^ = 0.0893, *R*^2^*change* = 0.0730), followed by Model 4 (*R*^2^ = 0.1422, *R*^2^*change* = 0.0695); the main factors were socioeconomic factors (urban *vs.* rural, age, sex, ethnicity, and education) and blood indicators.

To test the robustness of these results, a sensitivity analysis was performed using Cox proportional hazards regression analysis (see Supplementary Table 2). The results showed that the hs-CRP groups were significantly and positively correlated with the incidence of hypertension (*p* < 0.05). Compared with the CRP low-risk group, participants in the hs-CRP high-risk group had a 36.4% higher risk of hypertension after adjustment for all covariates (hazard ratio: 1.364, 95% CI: 1.006–1.849; *p* = 0.03).

### 3.4. AUC for Risk Factors for Predicting Incident Hypertension

**Figure 3:**
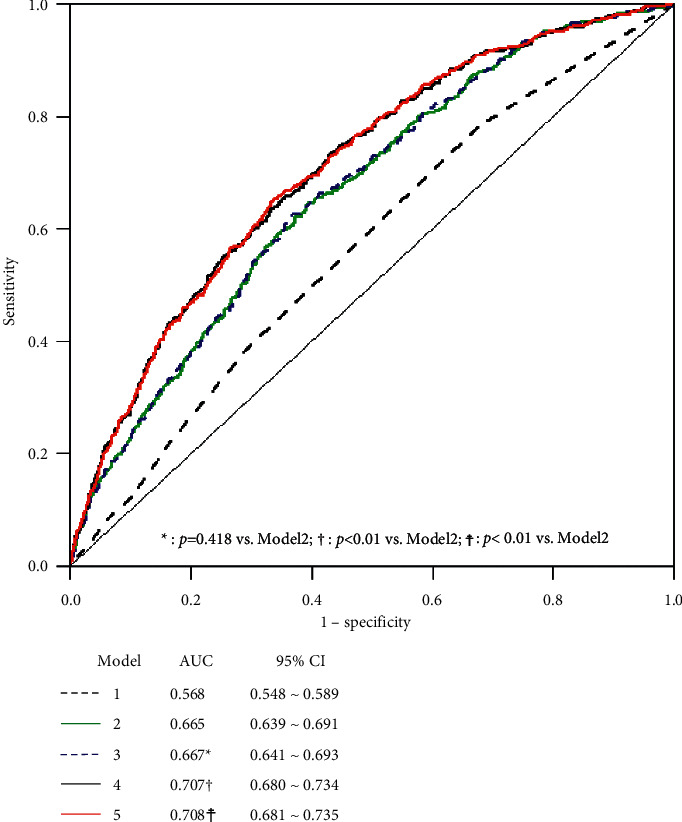
ROC curves for various models predicting incident hypertension.

The prediction model (model 1) based on hs-CRP yielded an AUC of 0.568 (Figure 3). Based on the conventional risk factors (gender, age, urban and rural, body mass index, smoking, drinking, and exercise), the prediction model (model 2) yielded an AUC of 0.665. Combining with the conventional risk factors and hs-CRP, the prediction model (model 3) yielded an AUC of 0.667 (95% CI: 0.680–0.734) compared to model 2 (*p*=0.418). Adding the high-risk factors (SBP, DBP, and WC) to prediction model (model 4) increased the AUC to 0.707 (95% CI: 0.680–0.734) compared to model 2 (*p* < 0.001). Combining with hs-CRP, the conventional risk factors, and high-risk factors, the prediction model (model 5) increased the AUC to 0.708 (95% CI: 0.681–0.735) compared to model 2 (*p* < 0.001).

## 4. Discussion

This is the first nationwide, large cohort study in China to examine the association between CRP levels and risk of hypertension. Our results show a weak positive association between CRP levels and future incidence of hypertension in apparently healthy, normotensive Chinese adults. The combination of hs-CRP with baseline BP and waist circumference (WC) had a higher predictive value for hypertension (AUC: 0.708), but the predictive value was limited.

Several epidemiological studies have explored the association between CRP levels and the risk of hypertension, but there is no consistent conclusion regarding the predictive ability of CRP [7, 8, 14, 15, 17, 18]. Most previous studies have demonstrated a positive association between CRP levels and the risk of hypertension in different populations, which is consistent with our findings; however, not all studies have demonstrated that CRP predicts the occurrence of hypertension accurately. For example, an analysis of a cohort of 795 diabetic patients exploring the risk of hypertension suggested that serum hs-CRP was not associated with the development of hypertension [18]. This may be due to the difference in the study population; all the positive correlation studies between CRP levels and the risk of hypertension were conducted in the general population and did not examine the relationship with hypertensive status in a diabetic cohort [32]. C-reactive protein was not found to be associated with elevated blood pressure in the British Women's Heart and Health Study [33] or in younger adults [10]. One meta-analysis [34] showed a significant association between hs-CRP and the development of hypertension (relative risk [RR] = 1.72, 95% CI: 1.38–2.07), as observed in a US population-based study, but not in Asian populations (RR = 1.03, 95% CI: 0.95–1.12). Also, the association was not significant in studies that controlled for BMI. This may be due to differences in the set of factors included as confounders in US and Asian studies. Indeed, few of the US studies in the analysis of hs-CRP controlled for measures of adiposity, and this may have resulted in an overestimate of the effect of this marker. Notably, even without adjusting for measures of adiposity, our results also show a weak positive association between hs-CRP levels and future incidence of hypertension, which is significantly lower than the strong positive association seen in Europe and the United States. Ethnicity may be a possible reason for the discrepancy in the conclusions. It is worth noting that Asians tend to have lower CRP concentrations and lower BMI, which may be confounding factors for CRP predicting the occurrence of hypertension [34]. A possible mechanism by which high CRP levels lead to hypertension may be that increased CRP levels may increase blood pressure by decreasing nitric oxide production in endothelial cells, leading to vasoconstriction [35, 36]. Meanwhile, CRP has also been reported to have proatherogenic properties by upregulating the expression of angiotensin type I receptors, thereby affecting the renin-angiotensin system and participating in the pathogenesis of hypertension [37].

Cross-sectional analysis of CRP with relevant covariates found that CRP group correlated most strongly with WC, baseline BP, age, and TG, while univariate analysis of baseline covariates with incidence of hypertension found that baseline BP, age, and WC contributed more to the risk of incident hypertension: systolic BP (*R*^2^ = 0.076), age (*R*^2^ = 0.063), diastolic BP (*R*^2^ = 0.052), WC (*R*^2^ = 0.039), and BMI (*R*^2^ = 0.024). Among the longitudinally adjusted models, models 2 and 4 showed the greatest variation in contribution, involving factors that included mainly age and baseline BP. In addition in model 5, WC and BMI interfered with the relationship of CRP to the incidence of hypertension. As a modifiable factor, the association of WC and BMI with hypertension has been subject to extensive research. A study by Carba et al. supports the notion that WC is strongly associated with hypertension, showing a 5% increase in the risk of hypertension in nonoverweight women for every 1 cm increase in WC after adjustment for age and other confounders [38]. Previous studies also showed that WC was predictive of the development of hypertension in Hispanic participants [39] and Asians [40]. It has also been shown that CRP mediates the relationship between BMI and hypertension [41]. Our study found that the relationship between CRP and hypertension was attenuated after controlling for WC and BMI. Meanwhile, based on clinical practice, the conventional risk factors are prioritized; the marginal benefit of including hs-CRP in risk stratification for hypertension in this study was attenuated (*p* > 0.05). Although, combined with baseline BP and WC, hs-CRP had a higher predictive value for hypertension, the overall AUC is still not impressive; therefore, the hs-CRP predictive value was limited.

The strengths of this study include the nationwide, prospective follow-up design, and adjustment for the main associated variables and having moderate reference value for clinical identification and prevention of risk factors for hypertension. In addition to the common weaknesses of observational studies and the interference of unknown factors, other limitations should be considered. Firstly, despite careful covariate adjustment in this observational study, residual confounding by unavailable dietary, certain medical conditions variables or metabolic factors may persist. Secondly, limited by the database, this study did not assess the use of drugs that lower CRP levels, such as statins; however, these drugs are unlikely to be used by participants with low CRP levels. Thirdly, due to the availability of data, we only used the 2009 biomarker data, but the intrinsic associations between CRP and hypertension are not affected by economic and social development. Moreover, limited by the database, the timeframe of 6 years is relatively short when observing total lifespan; younger population in this cohort will unlikely develop hypertension in the next 6 years but may still have a higher risk of future hypertension; therefore, they may require a longer timeframe to confirm in the future.

Our findings have several clinical implications. First, CRP may serve as a biomarker for predicting the risk of hypertension. However, our data suggest that CRP itself does not adequately predict hypertension and should be used in combination with other risk markers (e.g., baseline BP, age, WC, and TG). Second, there is a positive correlation between CRP levels and future incidence of hypertension; as such, targeting inflammatory pathways may be a potential avenue for the prevention or treatment of hypertension.

In conclusion, this study revealed a weak positive association between CRP levels and future incidence of hypertension in the Chinese population. We also showed that single hs-CRP values are less predictive than serial combined measurements. Therefore, to adequately predict hypertension, CRP should be used in combination with other high-risk markers, especially baseline BP, age, WC, and TG.

## Figures and Tables

**Table 1:** Characteristics of the participants from the 2009 CHNS according to the hs-CRP classification.

Variable	0 ≤ hs-CRP < 1	1 ≤ hs-CRP < 3	3 ≤ hs-CRP < 10	*p*
No. of participants	1135	2015	644	
Han nationality, *n* (%)	1000 (88.11)	1750 (86.85)	567 (88.04)	0.78
Countryside, *n* (%)	816 (71.89)	1420 (70.47)	456 (70.81)	0.54
Male, *n* (%)	463 (40.90)	958 (47.69)	275 (42.90)	0.11
Age (years)	43.74 ± 12.46	48.61 ± 12.69	50.51 ± 13.82	<0.01
Sleep duration, *n* (%)				
≤6h	91 (8.02)	229 (11.36)	71 (11.02)	0.39
6<h ≤ 8	791 (69.69)	1354 (67.20)	418 (67.90)	
>8 h	253 (22.29)	432 (21.44)	155 (24.07)	
BMI (kg/m^2^), *n* (%)				
<18.5	142 (12.51)	141 (7.00)	58 (9.01)	<0.01
18.5 ≤ BMI <24	766 (67.49)	1110 (55.09)	295 (45.81)	
≥24	227 (20.00)	764 (37.92)	291 (45.19)	
WC (cm)	77.61 ± 8.78	82.19 ± 9.40	84.36 ± 10.35	<0.01
Educational level, *n* (%)				
Primary school or below	393 (34.75)	844 (42.05)	281 (43.84)	<0.01
Middle school	607 (53.67)	936 (46.64)	303 (47.70)	
Technical or vocational degree	80 (7.07)	127 (6.33)	39 (6.08)	
College and above	51 (4.51)	100 (4.98)	18 (2.81)	
Glucose (mg/dL)	90.39 ± 13.84	95.16 ± 23.21	99.04 ± 28.07	<0.01
TG (mg/dL)	110.17 ± 87.92	147.63 ± 133.52	169.39 ± 143.62	<0.01
TC (mg/dL)	176.26 ± 33.98	187.57 ± 36.44	195.05 ± 39.52	<0.01
HDL-C (mg/dL)	59.14 ± 17.39	55.51 ± 16.87	53.49 ± 19.83	<0.01
LDL-C (mg/dL)	106.07 ± 31.29	114.91 ± 35.48	119.22 ± 37.58	<0.01
Total energy intake (kcal/day)	2133.42 (1754.86–2580.63)	2135.38 (1722.36–2590.18)	2042.47 (1722.58–2395.13)	<0.01
Smoking status, *n* (%)	300 (26.48)	666 (33.12)	194 (30.27)	0.02
Alcohol consumption, *n* (%)	359 (31.69)	701 (34.86)	207 (32.29)	0.52
Physical activity (MET-h/week)	140.00 (80.00–248.00)	142.13 (80.00–245.00)	146.00 (80.00–245.00)	0.74
Systolic BP (mm Hg)	114.01 ± 11.61	116.99 ± 10.99	117.98 ± 10.71	<0.01
Diastolic BP (mm Hg)	74.37 ± 7.92	76.42 ± 7.32	76.45 ± 7.15	<0.01
HOMA-IR	1.94 (1.36–2.86)	2.25 (1.55–3.35)	2.53 (1.75–3.96)	<0.01

Descriptive analyses of continuous variables were analyzed by means ± standard deviations (SD) or medians (interquartile range) were estimated, and categorical variables were described by number (percentage). Analysis of variance (ANOVA) or Kruskal–Wallis test was used for continuous variables, and chi-square test was used for categorical variables. CHNS: China Health and Nutrition Survey; hs-CRP: high-sensitivity C-reactive protein; BMI: body mass index; WC: waist circumference; BP: blood pressure; TG: total triglyceride; TC: total cholesterol; HDL-C: high-density lipoprotein cholesterol; LDL-C: low-density lipoprotein cholesterol; MET: metabolic equivalent; HOMA-IR: the homeostasis model assessment of insulin resistance.

**Table 2:** Correlation of hs-CRP with relevant covariates in the 2009 CHNS.

Variable	hs-CRP		
	*r*	95% CI	*p*
Systolic blood pressure	0.133	(0.099, 0.167)	<0.001
Age	0.189	(0.158, 0.219)	<0.001
Diastolic blood pressure	0.104	(0.071, 0.389)	<0.001
Waist circumference	0.243	(0.212, 0.273)	<0.001
BMI	0.186	(0.155, 0.216)	<0.001
Educational level	−0.067	(−0.098, −0.035)	<0.001
Total triglyceride	0.184	(0.153, 0.215)	<0.001
Glucose	0.141	(0.109, 0.172)	<0.001
Area	−0.011	(−0.042, −0.021)	0.515
Han nationality	−0.005	(−0.037, 0.026)	0.735
Total cholesterol	0.211	(0.180, 0.241)	<0.001
Alcohol consumption	0.012	(−0.019, 0.044)	0.447
Sex	−0.028	(−0.061, 0.003)	0.076
Physical activity	−0.003	(−0.043, 0.037)	0.872
Sleep duration	−0.014	(−0.045, 0.018)	0.404
Smoking status	0.041	(0.009, 0.073)	0.011
HDL-C	−0.154	(−0.184, −0.122)	<0.001
Total energy intake	−0.041	(−0.073, −0.008)	0.013
HOMA-IR	0.152	(0.121, 0.183)	<0.001

CRP, BMI, and sleep duration were grade variables; BP, age, waistline, TC, glucose, TG, HDL-C, total energy intake, and HOMA-IR were continuous variables; educational level, area, sex, Han nationality, alcohol consumption, and smoking status were categorical variables. CHNS: China Health and Nutrition Survey; hs-CRP: high-sensitivity C-reactive protein; BMI: body mass index; HDL-C: high-density lipoprotein cholesterol; HOMA-IR: the homeostasis model assessment of insulin resistance; CI: confidence interval.

**Table 3:** Univariate analysis of the incidence of hypertension and its contribution.

Variable	b	SE	*p*	OR	95% CI	*R* ^2^
hs-CRP (ref = 0 ≤ hs-CRP < 1)						
1 ≤ hs-CRP < 3	0.489	0.093	<0.001	1.630	(1.359, 1.956)	0.016
3 ≤ hs-CRP < 10	0.679	0.116	<0.001	1.971	(1.570, 2.476)	
Systolic blood pressure	0.051	0.004	<0.001	1.052	(1.044, 1.061)	0.076
Age	0.038	0.003	<0.001	1.039	(1.032, 1.045)	0.063
Diastolic blood pressure	0.062	0.006	<0.001	1.065	(1.052, 1.078)	0.052
Waist circumference	0.039	0.004	<0.001	1.040	(1.032, 1.048)	0.039
BMI (ref ≤ 18.5)						
18.5 ≤ BMI < 24	0.168	0.151	0.264	1.183	(0.880, 1.591)	0.024
BMI ≥ 24	0.754	0.153	<0.001	2.127	(1.575, 2.874)	
Educational level (ref = primary school or below)						
Middle school	−0.456	0.080	<0.001	0.634	(0.542, 0.741)	0.018
Technical or vocational degree	−0.719	0.178	<0.001	0.487	(0.343, 0.691)	
College and above	−0.755	0.214	<0.001	0.470	(0.309, 0.715)	
Total cholesterol	0.006	0.001	<0.001	1.007	(1.005, 1.009)	0.017
LDL-C	0.006	0.001	<0.001	1.006	(1.004, 1.008)	0.013
Glucose	0.008	0.001	<0.001	1.008	(1.005, 1.011)	0.009
Area (ref = urban)	0.388	0.088	<0.001	1.475	(1.241, 1.753)	0.008
Nationality (ref = Han)	0.508	0.129	<0.001	1.661	(1.290, 2.139)	0.006
Total triglyceride	0.001	0.001	<0.001	1.001	(1.001, 1.002)	0.005
Alcohol consumption (ref = yes)	−0.135	0.079	0.090	0.874	(0.747, 1.021)	0.001
Sex (ref = male)	−0.119	0.076	0.110	0.887	(0.764, 1.031)	0.001
Sleep duration (ref = 6 < h ≤ 8)						
≤6 h	0.107	0.124	0.391	1.113	(0.872, 0.420)	<0.001
≥9 h	−0.001	0.093	0.986	0.098	(0.831, 1.199)	
Smoking status (ref = yes)	−0.053	0.082	0.519	0.949	(0.808, 1.114	<0.001
HDL-C	−0.001	0.002	0.740	0.999	(0.995, 1.004)	0.000
HOMA-IR	<0.001	<0.001	0.889	1.001	(0.987, 1.105)	0.000

BMI: body mass index; hs-CRP: high-sensitivity C-reactive protein; HDL-C: high-density lipoprotein cholesterol; LDL-C: low-density lipoprotein cholesterol; HOMA-IR: the homeostasis model assessment of insulin resistance; SE: standard error; OR: odds ratio; CI: confidence interval.

**Table 4:** Multivariable-adjusted odds ratios of hypertension according to categories of hs-CRP in follow-up studies from 2009 to 2015 (*n* = 3794).

Total	0 ≤ hs-CRP < 1	1 ≤ hs-CRP < 3	3 ≤ hs-CRP < 10	*p*	*R* ^2^	*R* ^2^ *change* ^#^
Patient/total participants	200/1135	521/2015	191/644			
Crude OR (95% CI)^1^	Ref	1.630 (1.359, 1.956)	1.971 (1.570, 2.476)	<0.001	0.0163	—
Adjusted OR (95% CI)^2^	Ref	1.401 (1.159, 1.690)	1.572 (1.240, 1.992)	<0.001	0.0893	0.073
Adjusted OR (95% CI)^3^	Ref	1.473 (1.163, 1.866)	1.571 (1.145, 2.154)	0.001	0.0727	−0.016
Adjusted OR (95% CI)^4^	Ref	1.359 (1.037, 1.782)	1.621 (1.137, 2.310)	0.005	0.1422	0.069
Adjusted OR (95% CI)^5^	Ref	1.279 (0.793, 1.683)	1.462 (1.018, 2.101)	0.030	0.1501	0.008

^1^Model 1: original model without any adjustments; ^2^model 2: adjusted for place of residence, age, gender, nationality, and education; ^3^model 3: adjusted as for model 2 plus smoking status, alcohol consumption, sleep duration, total energy intake, and physical activity; ^4^model 4: adjusted as for model 3 plus glucose, total triglyceride, total cholesterol, and high-density lipoprotein cholesterol; ^5^model 5: adjusted as for model 4 plus body mass index and waist circumference. #: *R*^2^ change = *R*^2^(model (*A*+1)) − *R*^2^ (model(*A*)). hs-CRP: high-sensitivity C-reactive protein; OR: odds ratio; CI: confidence interval.

## Data Availability

Our study relied on data from CHNS. The datasets using during the current study are available at https://www.cpc.unc.edu/projects/china.

## Supplementary Materials

Supplementary Materials Supplementary Table 1: collinearity between baseline variables from the 2009 CHNS. Supplementary Table 2: multivariable-adjusted hazard ratios (HR and 95% CI) of hypertension according to categories of hs-CRP in follow-up studies from 2009 to 2015. Supplementary Figure 1: the box plots of CRP distribution.
