# Supplementary material for: C-Reactive Protein Levels in relation to Incidence of Hypertension in Chinese Adults: Longitudinal Analyses from the China Health and Nutrition Survey
Source: Int J Hypertens. 2021 Dec 10;2021:3326349. doi: 10.1155/2021/3326349 (PMC8683184; doi:10.1155/2021/3326349)
Supplement: Supplementary Materials — Supplementary Table 1: collinearity between baseline variables from the 2009 CHNS. Supplementary Table 2: multivariable-adjusted hazard ratios (HR and 95% CI) of hypertension according to categories of hs-CRP in follow-up studies from 2009 to 2015. Supplementary Figure 1: the box plots of CRP distribution. [file 3326349.f1.zip › Supplementary Tables.docx]

**Supplementary Table 1** Collinearity between baseline variables from the 2009 CHNS.

| Variables | b | SE | t -value | P | Tolerance^*^ | VIF |
| --- | --- | --- | --- | --- | --- | --- |
| Intercept | -1.32622 | 0.16403 | -8.09 | <.0001 | . | . |
| hs-CRP | 0.02943 | 0.01411 | 2.09 | 0.0371 | 0.89501 | 1.11731 |
| Educational level | -0.01558 | 0.01340 | -1.16 | 0.2451 | 0.75311 | 1.32782 |
| age | 0.00395 | 0.00091 | 4.33 | <.0001 | 0.73981 | 1.35169 |
| urban and rural | 0.06133 | 0.02322 | 2.64 | 0.0083 | 0.89091 | 1.12245 |
| gender | 0.02716 | 0.02855 | 0.95 | 0.3416 | 0.38978 | 2.56554 |
| Nationality | 0.03034 | 0.02545 | 1.19 | 0.2334 | 0.95741 | 1.04448 |
| sleep duration | 0.02999 | 0.01700 | 1.76 | 0.0778 | 0.96901 | 1.03198 |
| Smoking status | -0.01173 | 0.02648 | -0.44 | 0.6577 | 0.49986 | 2.00056 |
| Alcohol consumption | 0.05832 | 0.02317 | 2.52 | 0.0119 | 0.62305 | 1.60501 |
| Total energy intake | -0.00001 | 0.00001 | -0.30 | 0.7631 | 0.89073 | 1.12267 |
| Physical_activity | -0.00008 | 0.00008 | -0.97 | 0.3301 | 0.90689 | 1.10267 |
| Systolic BP | 0.00543 | 0.00111 | 4.89 | <.0001 | 0.53401 | 1.87262 |
| Diastolic BP | 0.00289 | 0.00164 | 1.77 | 0.0773 | 0.54046 | 1.85029 |
| TC | -0.00109 | 0.00061 | -1.80 | 0.0728 | 0.16189 | 6.17710 |
| TG | 0.00003 | 0.00012 | 0.28 | 0.7831 | 0.36815 | 2.71628 |
| HDL-C | 0.00027 | 0.00078 | 0.35 | 0.7300 | 0.54970 | 1.81916 |
| LDL_C^a^ | 0.00129 | 0.00056 | 2.30 | 0.0216 | 0.19941 | 5.01489 |
| glucose | 0.00058 | 0.00052 | 1.10 | 0.2704 | 0.67151 | 1.48918 |
| HOMA-IR | -0.00290 | 0.00160 | -1.81 | 0.0709 | 0.78674 | 1.27107 |
| BMI | 0.00081 | 0.01960 | 0.04 | 0.9673 | 0.59506 | 1.68051 |
| WC | 0.00306 | 0.00126 | 2.42 | 0.0156 | 0.53948 | 1.85364 |

^*^: It is considered that there is collinearity between variables when tolerance ≤ 0.2 or VIF ≥10. a: there is collinearity between TC and LDL_C, and LDL_C will be deleted when analyzed. CHNS: China Health and Nutrition Survey; hs-CRP: high sensitivity C-reactive protein; BMI: body mass index; WC: waist circumference; BP: blood pressure; TG: total triglyceride; TC: total cholesterol; HDL-C: high-density lipoprotein cholesterol; LDL-C: low-density lipoprotein cholesterol; HOMA-IR: The homeostasis model assessment of insulin resistance; VIF: variance inflation factor.

**Supplementary Table 2** Multivariable - adjusted hazard ratios (HR and 95% CI) of hypertension according to categories of hs-CRP in follow up studies from 2009 to 2015 (n =3794).

| Total | 0 ≤ hs-CRP < 1 | 1 ≤ hs-CRP < 3 | 3 ≤ hs-CRP < 10 | *p-trend* |
| --- | --- | --- | --- | --- |
| Patient / total participants | 200/1135 | 521/2015 | 191/644 |  |
| Crude HR (95% CI) ^1^ | Ref | 1.497 (1.272, 1.762) | 1.722 (1.412, 2.099) | <0.01 |
| Adjusted HR (95% CI) ^2^ | Ref | 1.315 (1.115, 1.551) | 1.409 (1.151, 1.725) | <0.01 |
| Adjusted HR (95% CI) ^3^ | Ref | 1.392 (1.130, 1.716) | 1.442 (1.098, 1.894) | <0.01 |
| Adjusted HR (95% CI) ^4^ | Ref | 1.303 (1.032, 1.646) | 1.479 (1.097, 1.994) | 0.01 |
| Adjusted HR (95% CI) ^5^ | Ref | 1.240 (0.979, 1.569) | 1.364 (1.006, 1.849) | 0.03 |

1. Model 1: original model without any adjustments; 2-Model 2: adjusted for place of residence, age, gender, nationality, education; 3-Model 3: adjusted as for model 2 plus smoking status, alcohol consumption, sleep duration, total energy intake, physical activity; 4-Model 4: adjusted as for model 3 plus glucose, total triglyceride, total cholesterol, high-density lipoprotein cholesterol; 5-Model 5: adjusted as for model 4 plus body mass index and waist circumference. hs-CRP: high sensitivity C-reactive protein; HR: hazard ratios; CI: confidence interval
