# Supplementary figures and images for: C-Reactive Protein Levels in relation to Incidence of Hypertension in Chinese Adults: Longitudinal Analyses from the China Health and Nutrition Survey
Source: Int J Hypertens. 2021 Dec 10;2021:3326349. doi: 10.1155/2021/3326349 (PMC8683184; doi:10.1155/2021/3326349)

## Slide 1
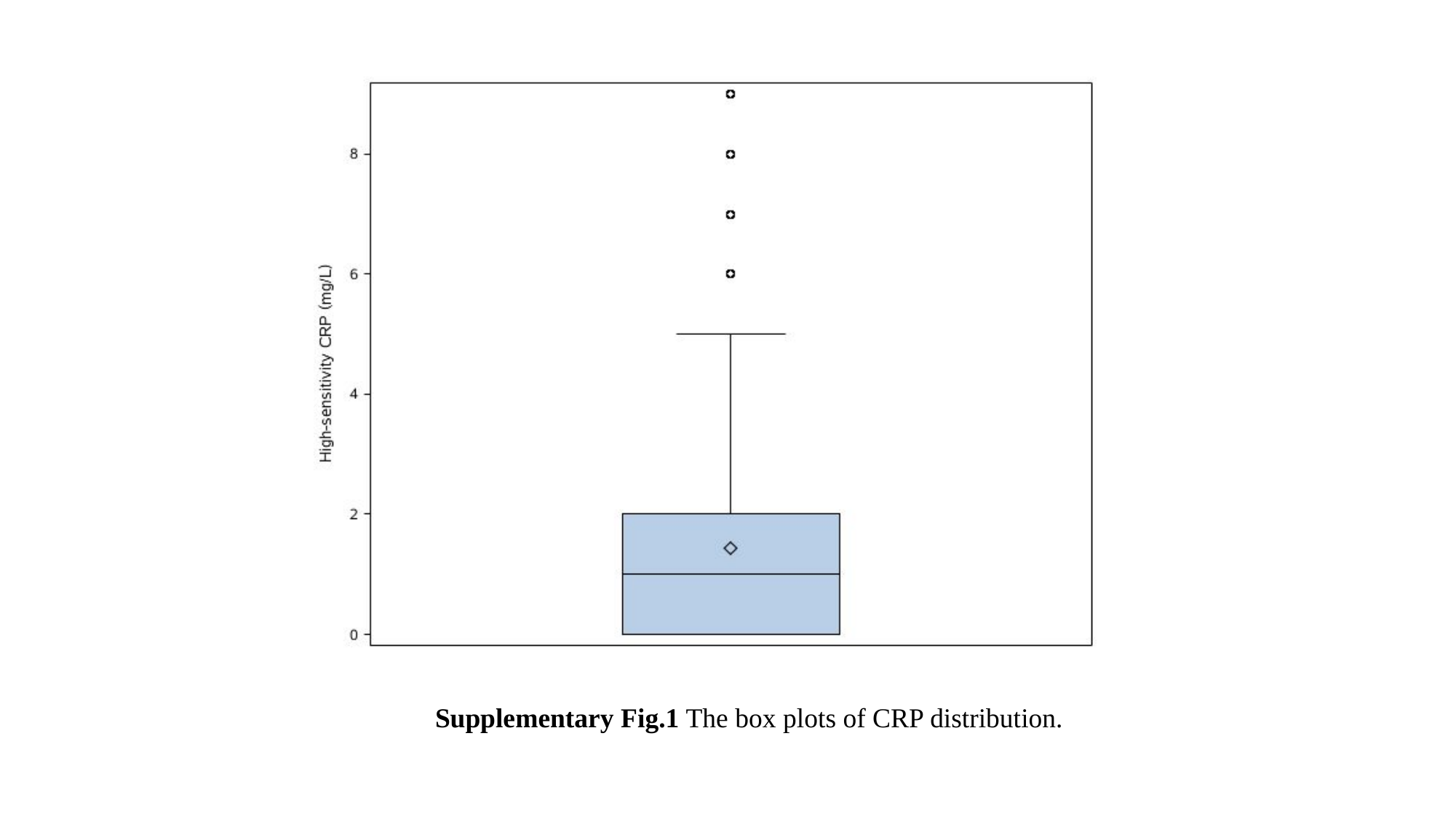

Supplementary Fig.1 The box plots of CRP distribution.

Supplement: Supplementary Materials — Supplementary Table 1: collinearity between baseline variables from the 2009 CHNS. Supplementary Table 2: multivariable-adjusted hazard ratios (HR and 95% CI) of hypertension according to categories of hs-CRP in follow-up studies from 2009 to 2015. Supplementary Figure 1: the box plots of CRP distribution. [file 3326349.f1.zip › Supplementary fig 1.pptx]
